# Sultanate of Oman: building a dental workforce

**DOI:** 10.1186/s12960-015-0037-z

**Published:** 2015-06-22

**Authors:** Jennifer E Gallagher, Sivakumar Manickam, Nairn HF Wilson

**Affiliations:** Division of Population and Patient Health, King’s College London Dental Institute at Guy’s, King’s College and St Thomas’s Hospitals, Denmark Hill Campus, Bessemer Road, London, SE5 9RS UK; Oman Dental College, P.O. Box 835, 116 Muscat, Sultanate of Oman; King’s College London Dental Institute, Guy’s Campus, London, SE1 9RT UK

**Keywords:** Oman, Dental students, Workforce, Human resources for health, Dental professionals, Dental team, Dentist, Dental hygienist, Dental therapist, Workforce planning

## Abstract

**Background:**

A medium- and long-term perspective is required in human resource development to ensure that future needs and demands for oral healthcare are met by the most appropriate health professionals. This paper presents a case study of the Sultanate of Oman, one of the Gulf States with a current population of 3.8 million, which has initiated dental training through the creation of a dental college.

**Objectives:**

The objectives of this paper are first to describe trends in the dental workforce in Oman from 1990 to date and compare the dental workforce with its medical counterparts in Oman and with other countries, and second, to consider future dental workforce in the Sultanate.

**Methods:**

Data were collected from published sources, including the Ministry of Health (MoH), Ministry of Manpower (MoM), and Ministry of National Economy (MoNE)-Sultanate of Oman; the World Health Organization (WHO); World Bank; and the Central Intelligence Agency (CIA). Dentist-to-population ratios were compared nationally, regionally and globally for medicine and dentistry. Dental graduate outputs were mapped onto the local supply. Future trends were examined using population growth predictions, exploring the expected impact in relation to global, regional and European workforce densities.

**Results:**

Population growth in Oman is increasing at a rate of over 2% per year. Oman has historically been dependent upon an expatriate dental workforce with only 24% of the dentist workforce Omani in 2010 (*n* = 160). Subsequent to Oman Dental College (ODC) starting to qualify dental (BDS) graduates in 2012, there is an increase in the annual growth of the dentist workforce. On the assumption that all future dental graduates from ODC have an opportunity to practise in Oman, ODC graduates will boost the annual Omani dentist growth rate starting at 28% per annum from 2012 onwards, building capacity towards global (*n* = 1711) and regional levels (Gulf State: *n* = 2167) in the medium term.

**Conclusion:**

The output of dental graduates from Oman Dental College is improving the dentist-to-population ratio and helping the Sultanate to realize its aim of developing an Omani-majority dental workforce. The implications for retention of dentists and team training are discussed.

## Background

Dental professionals play a vital role in meeting the oral health needs and demands of individuals in society. It therefore is important to ensure that the workforce resource is appropriate to the population, particularly given the cost of training and a working life that can span four decades. This paper presents the case study of the Sultanate of Oman, one of the Gulf States in the Middle East, which forms part of the Gulf Cooperation Council (GCC).

Globally, there is a health workforce shortage [1,2] and in many regions of the world, including the Middle East, a dental workforce shortage [3,4]. Furthermore, there are dramatically marked inequalities in access to oral and dental care, while dental disease (namely caries and periodontal diseases) remains by far the most prevalent of the non-communicable disease globally [5-7]. Global movement of populations and dental health professionals presents a major challenge to planning for the future [8,9]; therefore, it is important that each country regularly examines its dental workforce needs to ensure that it is taking account of population changes, health needs, workforce numbers, skills and expectations. In an ideal world this would occur on a regular basis [10].

**Figure 1:**
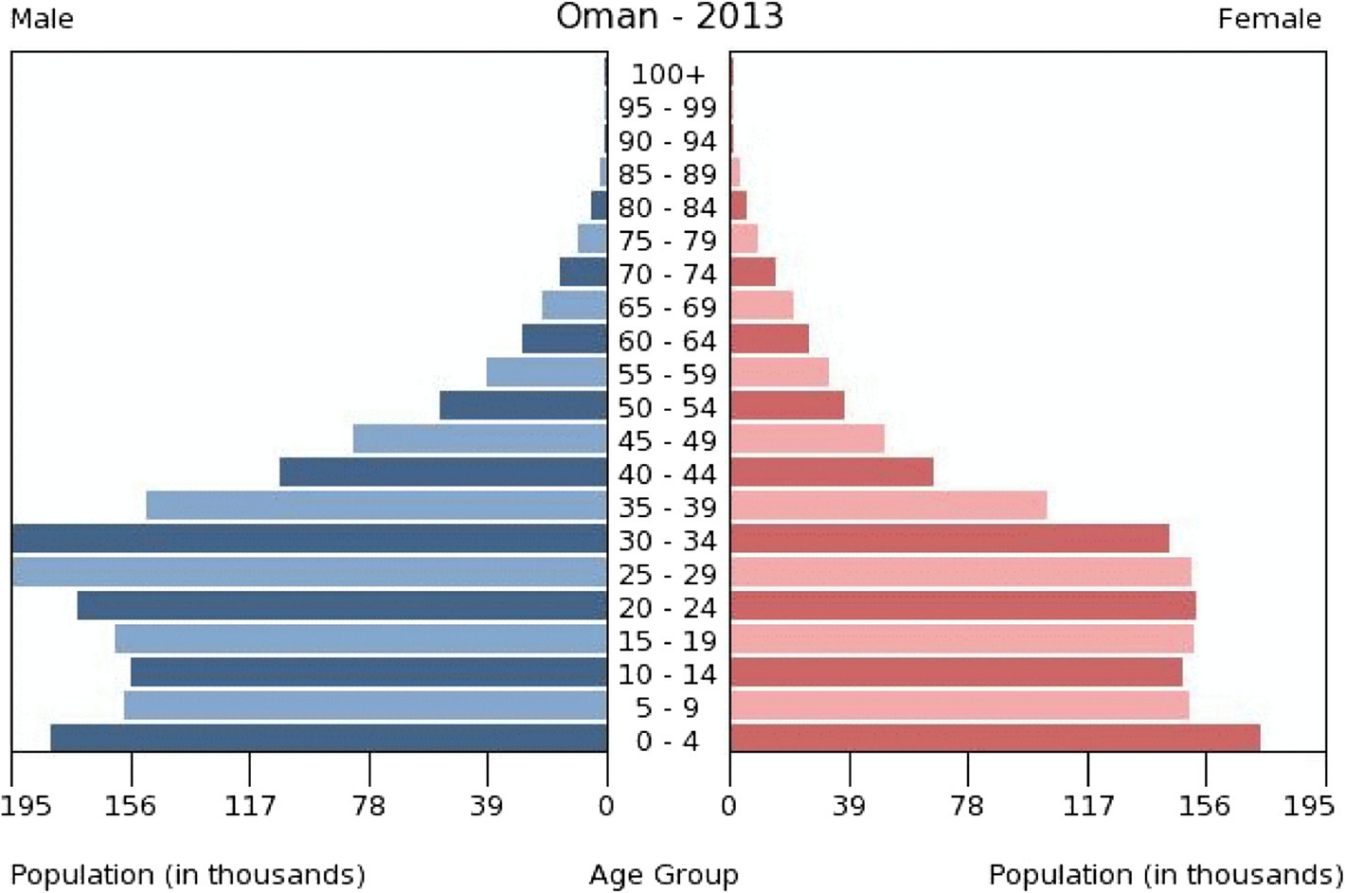
Age distribution of the Omani population *Source:* [13]

As countries recognize the importance of oral health for overall health and well-being, and the need for oral health care, so the education and training of dentists is facilitated. Oman, with a population approaching 4 million and a growth rate of over 2%, is one such case [11]. Dental training in Oman follows the European model [12]. A significant proportion of the population is expatriate. Figure 1 presents the age distribution of the Omani population during 2013 [13]; the data suggest that the 20–24-year age group comprised almost 41.7% of the total population; many of the males in particular will be expatriates attracted to work in Oman. Overall, the population is young but ageing; by 2050, it is anticipated that the population of Oman will exceed 7 million with the proportion of older people increasing significantly.

Oman has made a rapid transition to become a high-income country. This is reflected in improved population life expectancy, together with year-on-year increases in the prevalence of chronic non-communicable diseases, including diabetes and cardiovascular disease [14]. Population growth is high due to a high birth rate and the presence of expatriate workers, particularly males (Fig. 1). Infectious diseases have reduced markedly, but non-communicable diseases are high for this country, when compared with other Gulf States [15]. Oral diseases are non-communicable with common risk factors [16,17]. The oral health needs of the population in Oman are high. Almost all 6-year-olds (85%) have evidence of dental caries experience with an average of five teeth affected [18,19]; this level of dental caries experience is very high in relation to most countries [20]. Past surveys suggest that there are marked oral health differences within the country, with fluoridated Muscat having much reduced levels of dental caries, while other regions of the Sultanate have higher levels of disease [21,22].

Important dental public health developments have included the introduction of water fluoridation in the Muscat area, together with school oral health preventative programmes involving fissure sealants, tooth brushing and the application of fluoride [23]. Furthermore, the Ministry of Health has had a number of ongoing community initiatives for oral health in schools. These may be contributing to recent promising improvements in oral health amongst the 12-year-olds in the Sultanate [18]; the average dental caries experience score (decayed, missing and filled teeth = DMFT) having fallen from 2.5 in 1991 to 1.3 and affecting 51% of children in 2006 [18,19]. However, the effects of dental caries in the permanent dentition are cumulative and lifelong [22]. A recent local survey of adult health [18] acts as a reminder that dental caries experience in adults remains high. As a consequence, the oral health needs of the adult population will, for the foreseeable future, include the management of dental caries along with the maintenance and repair of heavily damaged and restored caries-prone dentitions. This suggests the need for a dentist workforce with a diverse range of skills, including good restorative and surgical skills, and highlights the importance of having a strong emphasis on oral health promotion.

As a developed country, Oman has placed great emphasis on developing its medical workforce and healthcare system [24,25]. The Sultanate has worked closely with the World Health Organization (WHO) to espouse the primary healthcare approach and address key public health issues. There have been significant successes in tackling, by way of example, infant mortality [15]. Oman has recognized the importance of improving human resources to achieve sustainability in its healthcare systems and has taken various steps that include achieving self-reliance and incorporating collaborative efforts from civil, armed forces, educational and private institutions to strengthen the country’s workforce [26].

Until recently, Omanis who wished to study dentistry had to train abroad, there having been no dental education available in the Sultanate. Thus, traditionally, Oman relied on expatriate dentists to form its dental workforce, together with a minority of overseas-qualified Omanis. With new policy encouraging the participation of the private sector in higher education, and the determination of its founding figures, Oman Dental College (ODC) [27] was established in 2006 and runs a 5-year BDS dental degree programme with an additional pre-dental year for the majority of those requiring preparation for the degree programme delivered in English. ODC remains the only dental school in Oman. It was established in consultation with the Ministries of Health and Higher Education and following approval of the Higher Education Council. ODC is regulated by the Ministry of Higher Education, and the majority of students have state bursaries.

Gallagher and Wilson [10] advocate the need for ongoing monitoring of the dental workforce, together with exploring future scenarios, recognizing that there are multiple pressures for change on the dental workforce: from demographic and epidemiological trends through social and economic change, political and policy to innovations in science and technology. They further elaborate that the sufficiency of a dental workforce is influenced by key stakeholders such as the government, universities, health service management, patients and the public.

The aggregate number of dentists in any country and their contribution to the workforce is vital to meet the need, and demand, for oral healthcare services within high-income countries with an established healthcare system. The purpose of the present study was to investigate the dentist workforce in Oman and to compare Oman’s dentist workforce data with global and regional norms. It is important to elucidate the future requirements of dentists in Oman, the Sultanate not yet having embraced the concept of the dental team, and to explore the extent to which these requirements can be met by ODC.

The objectives of this paper are to describe trends in the dentist workforce in Oman from 1990 to date and compare workforce supply density with medicine nationally and dentistry regionally and globally, as well as examine dentist workforce requirements until 2020 based on population estimates.

## Methods

Dental workforce analysis, to inform planning, is an important strategic healthcare activity that takes account of, and seeks to reflect, changes and pressures appropriately [28]. The literature contains a range of approaches which represent modelling systems of increasing sophistication and complexity including moving from deterministic models where there is a predictable relationship between inputs and outputs through to stochastic models where chance and uncertainty are included. Broadly speaking, the models take account of need [29-31], supply [3,30,32-37] and demand [38,39] for care. Each has its strengths and weaknesses; however, models which integrate population needs and workforce supply are of benefit from a public health perspective as they take account of the population [38,40]. Various modelling tools are available to support and combine some or all of these approaches. They include econometric [41,42] to linear programme models [38,40,43] and alternatives such as systems dynamics and stochastic models which take account of uncertainty [30,44].

At their most basic, integrated models may be presented as supply density [45]. It is alternatively expressed as the number of dentists per 10 000 population as in the WHO annual statistics [33] or conversely the ratio of persons in the population per dentist [3]. Because of its simplicity, it is the most commonly used metric for workforce comparison and provides a ‘broad brush’ approach that may provide a helpful starting point for planners and policy makers, as it takes account of population growth as a proxy for need and demand. Where local data are available, within-country ratios can take account of geographical distributions such as urbanization [34,45]. Benchmarking of supply against similar countries can provide a useful basis for considering the workforce supply; however, consideration needs to be made that the need for care and use of services will vary according to population demography, health needs, service models and costs [45].

This paper has adopted a basic integrated model using dentist-to-population ratios, benchmarking supply to provide comparisons with other countries. This approach is straightforward, transparent and most appropriate based on locally available data on the population and the dental supply. Oman publishes annual data on its health workforce numbers including the numbers of Omani and expatriate dentists [14]. Its single dental school, ODC, has a set intake which ranges from about 50–65 per year. More sophisticated modelling requires robust data on human resources and other key variables which are not readily available in most countries [8,9]; Oman is no exception. Furthermore, the WHO has constantly used dentist density as a measure for describing the volume of oral healthcare professionals in different countries [33,46]. In addition, it enables consideration of trends over time in Oman. Such population estimates can be used as a baseline to inform future scenarios and can inform future workforce models, research and analysis. The development of more complex models will be appropriate as the workforce expands and increases in complexity when there is a need for debate on skills mix [38,40,43], and robust data are available to support workforce planning [8,9].

Population projections form the backbone of any workforce analysis as the main aim of the workforce is to serve the population [47], and population projections are available for Oman [24]. Future scenarios are commonly used to accommodate uncertainty and may explore alternative predicted or desired futures [37]. Given that Oman is a high-income country that has made great strides in developing its medical workforce, comparisons were examined in relation to regional and WHO Regional European averages, taking into account the new flows of graduates from ODC into the dental workforce:

- *Option 1*: dentist: population density equivalent to the level considered appropriate to meet future oral health needs across Europe [33].
- *Option 2*: dentist: population density to match the current GCC average [33].
- *Option 3*: dentist: population density to match the current global average [33].

The following assumptions underpin these scenarios. First, the addition of dental (BDS) graduates from ODC to the Omani dental workforce remains constant at circa 50, given that a number of ODC graduates will go abroad to work or study each year. Second, the current level of expatriates will be maintained by inflow to account for outflow, death and retirement. Third, population growth continues on its upward trajectory. Fourth, and finally, selection of dentistry as a career remains popular amongst prospective students and student numbers remain unchanged in the medium term.

## Results

Only 24% of the dentist workforce was Omani in 2010; this ranged from 53% Ministry of Health, 68% in non-MoH government sector which includes the military services and only 1% in the private sector [26].

**Figure 2:**
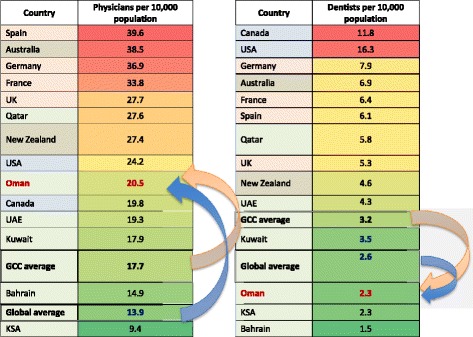
The relative position of Oman in physician and dentist density. *Source*: CIA [33]

WHO data provide comparisons of physician- and dentist-to-population ratios across the globe [33]. First, comparison within the Gulf States suggests that Oman’s physician density (20.5 per 10 000 population) is one of the best amongst GCC nations while dentist density (2.3 per 10 000 population) remains lower than the GCC average (3.2 per 10 000) and second worst in the region jointly with KSA [33], as presented in Fig. 2. Second, the dentist-to-population ratio in Oman is lower than the global average (2.6 per 10 000). Third, and finally, it is substantially below high-income countries such as the United Kingdom (UK; 5.3 per 10 000) and the United States of America (USA; 16.3 per 10 000).

**Figure 3:**
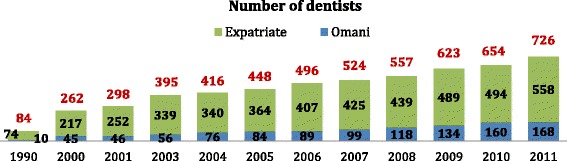
Trends in the number and nationality of dentists in Oman. *Source:* Ministry of Health [66]

**Figure 4:**
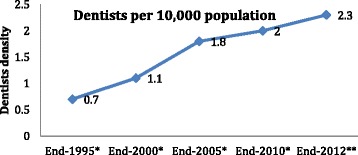
The growth of the dentist workforce in Oman, 1990–2012. *Source*: Ministry of Health and World Health Organization [18,33]

Looking at trends in the Oman dental workforce, there has been a moderate increase in the number of dentists observed during the 2000s (Figs. 3 and 4), a sharp increase observed from 2005 onwards in the dentist workforce in Oman, with a positive contribution from ODC since 2012 amounting to 47 and 56 graduates in 2012 and 2013, respectively, not all of whom have joined the workforce.

Considering the recent rise in the number of dentists in Oman bringing the dentist-to-population ratio closer to the global average of 1:3800, it is important to consider the short- to medium-term perspective, namely 2012–2020, in more detail. The expected increase in the number of Omani dentists should contribute to the total required number of dentists in Oman and bring down the expatriate growth rate to pave the way for further Omanisation.

**Figure 5:**
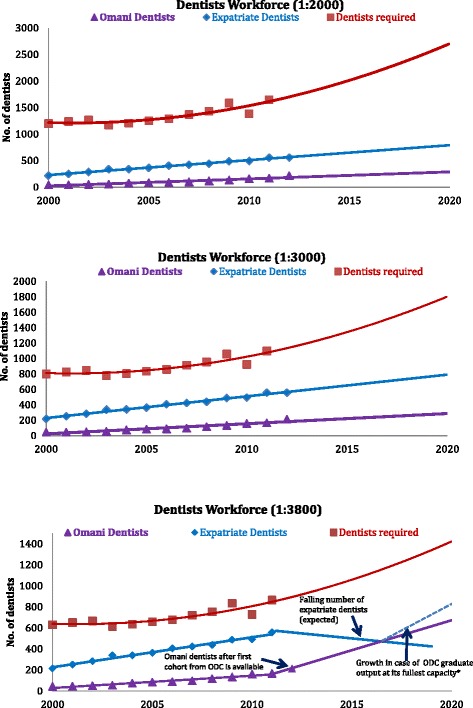
Dentist workforce capacity projections. **a** Dentist workforce capacity: projection at 1: 2000. **b** Dentist workforce capacity: projection at 1: 3000. **c** Dentist workforce capacity: projection at 1: 3800
- *Option 2*: dentist: population density to match the current GCC average (1:3000 or 3.2 per 10 000) will require circa 1500 dentists currently as presented in Fig. 5b, to achieve 2167 dentists by 2020.
- *Option 3*: dentist: population density to match the current global average (1:3800 or 2.6 per 10 000) will require circa 1000 dentists currently as presented in Fig. 5c, to achieve 1711 dentists by 2020.

- *Option 1*: dentist: population density equivalent to the level considered appropriate to meet future oral health needs across the WHO European region (1:2000 or 5 per 10 000) will require 1900 dentists currently as presented in Fig. 5a, with further increases to 3250 dentists by 2020.

With reference to the original feasibility study conducted by ODC prior to it being established, it is important to note several points. First, a reduction in the shortfall in dentists was expected, and second, that the shortfall would not be eliminated by the initial student intake and graduate output. Based on the latest published data, this feasibility study projection has been shown to be realistic. The model suggests that the projected increase in dental graduates from ODC from 2018 onwards will help to narrow the gap between the Omani dentist-to-population ratio and that of local GCC as well as moving towards high-income regions such as Europe. This is of course assumes that most ODC graduates are encouraged, and have the opportunity, to practice in Oman, and the number of expatriate dentists remains relatively constant.

## Discussion

The findings suggest that there is a need for more dentists to enter into the workforce locally to match global capacity and work towards the GCC dentist-to-population ratio of 1:3000, or 3.2 per 10 000. In 2010, Omanisation of the dentist workforce was just around one quarter (24%). To achieve the two local dental workforce goals, the retention in Oman of an annual graduate output of about 50 dentists from ODC will be vital, assuming population growth remains moderate to high. It is interesting to note that ODC’s 2003 feasibility study estimated that with an output of 39 graduates, there would be 213 Omani dentists available in the country by 2011 (ODC, 2003). However, MoH data suggest that as of 2011 there were only 168 Omani dentists [14], emphasizing the need to increase and retain the dental graduate output of ODC. It is suggested that the required increase in dental graduate output, with the desired Omanisation of the dental workforce, may be made by ODC.

It is clear, in terms of the physician: population ratio, that Oman is well above the global average [33], and ahead of many nations as affirmed by the WHO. However, in relation to the dentist-to-population ratio, Oman is not only lower than the local and global average but there are very few other nations with sound and effective healthcare systems that have a lower ratio. If oral health needs were low, there would be a rationale for accepting a reduced dentist-to-population ratio; however, needs are high, and the majority of disease in children appears to be unmet. Furthermore, the relatively high levels of dental caries experience historically in older children [21,22] mean that their needs in adulthood will continue as the disease is cumulative.

It is firmly acknowledged, by the authors, that a growth in dentists is not the ‘answer’ to meeting the needs of the population of Oman. There is an unquestionable need for primary prevention of oral and dental disease [18,21,22], delivered through a range of general public policy and community initiatives [48], and Oman as a country espouses an effective public health approach [24]; however, this must be supported by appropriately trained dental personnel with an emphasis on promoting health. This approach involves team working [49] and team training involving dental hygiene and/or dental therapists as well as dental nurses [50], in addition to community health workers promoting oral health. Furthermore, access to dental care should be equitable, and this will require a co-ordinated approach to ensure that intra-country planning and action ensures that dentists work across all areas of the country, rather than being concentrated in the capital city. Considering the high levels of dental caries experience in the Omani population, together with the cohort effect, whereby yesterday’s teenagers become today’s adults with significant accumulated caries experience (21,22), suggests a strong need for an increased dental workforce for many years to come – especially given the rapidly increasing life expectancy of Omanis, a workforce that could include the introduction of dental hygienists and/or therapists to Oman. Furthermore, from each cohort of ODC graduates, the number pursuing postgraduate studies and specialty training internationally may be anticipated to increase, given the limited opportunities for such career advancement locally. Thus, even if high-population growth is not maintained, the existing dental workforce capacity, including the anticipated numbers of dental graduates from ODC, will be insufficient to meet the population needs in the foreseeable future. Under such circumstances, it can be argued that not only should the dental graduate output of ODC be increased but, in addition, arrangements should be put in place to encourage ODC graduates to return to Oman to practice following the successful completion of postgraduate studies and training internationally. Alternatively, and possibly preferably, new opportunities should be created for ODC graduates to undertake postgraduate studies and training locally, thereby limiting a dental ‘skills drain’. Health workforce migration is a global challenge, and countries wishing to retain a workforce must consider the necessary factors to monitor and manage migration, particularly where it is state funded, while also recognizing the rights of healthcare workers [51].

### Strengths and limitations

It is well accepted that population and dental workforce projections depend on multiple factors, not all of which can be accounted for, as some influences are unexpected. Population projections depend on assumptions about mortality, fertility, base life expectancy and migration. Dental and dentist workforce projections are influenced by additional factors such as the economy, population demand for oral healthcare, government policy and the contribution of health professionals to workforce capacity. Naturally, any changes in these assumptions may affect these projections.

The tremendous decline in the infant mortality rate in Oman is contributing to population growth, and there is a large expatriate population of a potentially highly variable size working on a range of projects and developments [24]. Furthermore, the high proportion of young people suggests a high potential for future population growth, thus making absolute projections difficult. However, as Oman is currently exceeding its predicted population growth [47,52], and it is anticipated that there will continue to be a large expatriate population, the population predictions used in the present study for high- or medium-population growth are considered realistic. It is acknowledged that monitoring population growth is vital and as the dentist population expands, should it deviate from the anticipated trajectory, the assumptions in the model must be revisited. It will be important that all sections of the population have access to contemporary oral and dental care as the workforce expands.

Analysis of dentist-to-population ratios is a crude measure, because it does not take account of the needs of different sections of the population. Neither does it consider the complexity of supply issues including professional working patterns, particularly for younger dentists and females. In the United Kingdom and the United Arab Emirates, there is some evidence that graduates, both male and female, do not necessarily plan to work full time and some, irrespective of opportunities locally, will plan to travel and work or study overseas [53,54]. Thus, the dentist workforce estimates for Oman used in the present study are possibly an overestimate. Furthermore, whereas many of the expatriates are male, the majority of graduates from ODC are female, and their contribution to the workforce is as yet unclear. There is a suggestion from research in a neighbouring Gulf State that females expect to make similar levels of contribution to their male counterparts [53,54]. However, there is no evidence of the working pattern of dentists in the current workforce in Oman; research is required into these areas to provide better information for dentist workforce analysis and planning.

Given the current emphasis on training mid-level providers globally [55-57], such as dental hygienists and therapists, the rationale for including such members of the dental team in this model must be considered. First, the presence of significant disease levels, together with the affluence of Oman, means that dentists ± therapists will be required to provide the majority of care required; second, the next workforce development should ideally be dental nurses (otherwise known as dental surgery assistants) to ensure that greatest use is made of dental skills [10,58]. The need for mid-level providers should be explored in future workforce analyses working with a broad range of key stakeholders [59]. Such analyses should involve a joined up, collaborative approach by all relevant stakeholders in any future modelling developments.

### Future research and action

The data used for this analysis highlight the need for ongoing research to inform dental workforce planning; this must include consideration of the changing health needs of the population, a regular census of the workforce population [10], understanding of the career expectations of new entrants to the dental profession and the subsequent trajectory of careers. Thus, it will be important to explore the expectations of dental graduates, as from other dental schools [53,54,60-62], and monitor the trends in dental workforce stocks and flows for Oman nationally and regionally. This would allow for more detailed analysis and modelling as the workforce expands and consideration of how to address inequalities in oral health at the district level within the country [18,21,22].

A further aspect of the development of the dentist workforce which is beyond the scope of this paper is the need to develop a workforce of dental specialists. Oman presently only supports specialty training in oral and maxillofacial surgery (OMFS) locally, thus, as alluded to above, existing graduates seeking to train in dental specialties must do so abroad, joining a general drift in healthcare workforce personnel with no, or limited, career development opportunities nationally towards countries which offer specialization and postgraduate education. Early consideration should be given to developing postgraduate and specialist training programmes in Oman. According to international practice, such training programmes are best based in a dental school and hospital, thus providing a clinical academic environment.

Finally, it must be stressed that for an existing and future dental workforce to be effective, consideration needs to be given to the development of additional members of the team involving team training and care delivery [58]. As outlined above, early consideration should be given to establishing a substantive and co-ordinated programme of dental nurse training in Oman. There are moves in other countries such as the UK for dental nurses to be trained and registered with the General Dental Council [63-65]. Their numbers exceed those of dentists and they play an important role in support of quality oral healthcare provision and the protection of the public. To be fully effective in supporting the modern, safe clinical practice of dentistry, dental nurses should be trained together with dentists and other members of the dental team, with the volume of dental nurses exceeding dentists; the exact ratio will depend on workforce patterns. Overall, this highlights the need for consideration to be given to dental workforce development through the adoption of a wider dental team approach to future oral healthcare provision, which, in turn, will contribute to improved general health and well-being.

## Conclusion

This case study illustrates the benefits of workforce analysis to inform workforce development. The present analysis suggests that consideration should be given in Oman to embracing the output of dental graduates from the Sultanate’s new, and only, dental school to bring the dentist-to-population ration closer to the global (1:3800) or GCC ratio (1:3000), if not the level considered appropriate to meet future oral health needs across Europe (1:2000) and realize the Oman Ministry of Health aim to develop an Omani-majority, dental workforce. The discussion highlights the need for consideration to be given to dental workforce expansion through the adoption of a dental team approach to future oral healthcare provision and the introduction of postgraduate and specialty training programmes in Oman, all of which should be informed by further workforce development research. There is a pressing need in Oman to establish a dental nurse training programme to ensure that there are sufficient numbers of suitably trained dental nurses to support the safe, effective practice of dentistry in the Sultanate, followed by wider team development.

## Abbreviations

CIA: Central intelligence agency
GCC: Gulf cooperation council
ODC: Oman dental college
MoH: Ministry of health, Sultanate of Oman
MoM: Ministry of manpower, Sultanate of Oman
MoNE: Ministry of national economy, Sultanate of Oman
WHO: World health organization
